# New Primary Standard Set for Fine Particulate Matter

**DOI:** 10.1289/ehp.121-a74

**Published:** 2013-03-01

**Authors:** Bob Weinhold

**Affiliations:** Bob Weinhold, MA, has covered environmental health issues for numerous outlets since 1996. He is a member of the Society of Environmental Journalists.

Ambient concentrations of fine particulate matter (PM_2.5_) have dropped in recent years as a result of regulations set by the U.S. Environmental Protection Agency (EPA), and the agency anticipates this trend will continue.^1^ But hundreds of studies conducted since the regulations were last reviewed indicate that stricter rules still might be warranted to adequately protect the U.S. population against adverse cardiovascular, respiratory, and possibly other health effects associated with PM_2.5_.^2^ In December 2012 the EPA reduced the annual primary National Ambient Air Quality Standard (NAAQS) for PM_2.5_ to a level it anticipates will protect public health with an adequate margin of safety when combined with the existing 24-hour standard.^3^

The new primary annual standard of 12 µg/m^3^, down from the previous 15 µg/m^3^, falls at the low end of the 12- to 13-µg/m^3^ range the EPA proposed in June 2012.^4^ With the new standard, more than 44 million U.S. residents live in counties that would violate the standard if it were fully in effect today, based on 2009–2011 monitoring data.^5^^,^^6^ However, by 2020—the deadline for implementation—only seven monitored counties are expected to be in violation as a result of ongoing implementation of other regulations already in place, such as those targeting diesel engines, other onroad and offroad vehicles, waste incinerators, and coal-fired power plants.

It’s plausible that ambient concentrations of PM will be generally lower nationwide in 2020 than they are today, says Lorraine Gershman, director of environment, regulatory, and technical affairs with the American Chemistry Council (ACC), which represents some of the industries affected by the regulation. That, along with inconsistencies in findings on the health effects of particulate pollution, is why the ACC opposed lowering the standard.^7^

Primary standards are set to protect human health against acute and chronic effects, whereas secondary standards are intended to address a range of environmental impacts, such as climate effects, damage to materials, and visibility impairment. The previous annual primary PM_2.5_ standard of 15 µg/m^3^ was set in 1997.^8^ It was retained by the Bush administration in 2006, but in 2009 the U.S. Court of Appeals for the D.C. Circuit remanded the standard to the EPA and directed the agency to explain how it would protect public health with an adequate margin of safety as required under the Clean Air Act.^9^ The EPA attempted to address the court-identified shortcomings with this new 2012 regulation.

**Figure:**
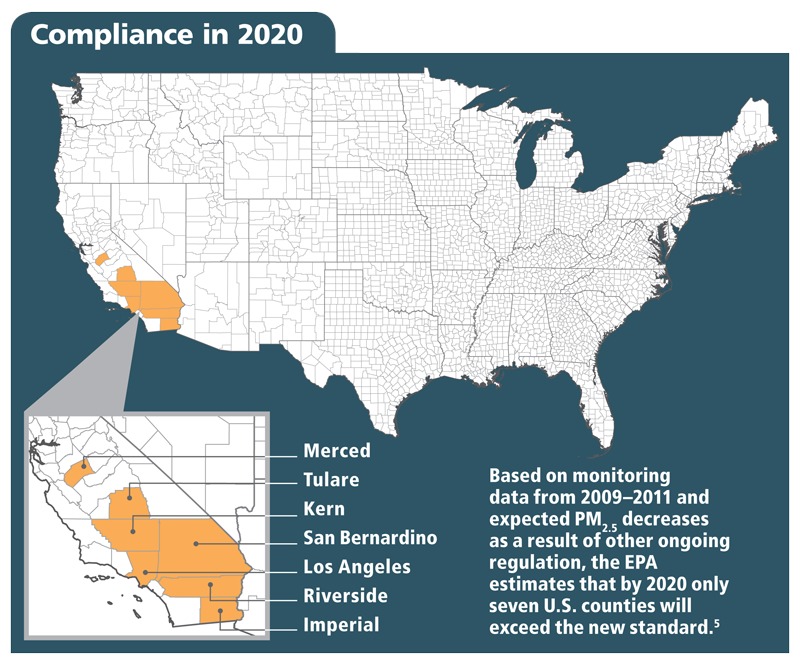


The 24-hour primary standard remains unchanged from the Bush administration concentration of 35 mg/m^3^, a threshold that wasn’t challenged in court. Also unchanged are the annual and 24-hour secondary standards, which remain at 15 mg/m^3^ and 35 mg/m^3^, respectively (although both were challenged in the post-2006 legal actions by various parties). The primary and secondary 24-hour standards for coarse particulate matter (PM_10_), established in 1987 and upheld by the court in its 2009 ruling, also remain unchanged at 150 µg/m^3^.

The agency estimates the only localities that will need to take action to meet the new set of PM standards will be seven counties in California, and possibly all or parts of some nearby counties. For these counties, which include some of California’s most heavily populated, the agency estimates the updated standards will lead to annual benefits of $4–9.1 billion in avoided health problems and premature deaths, with estimated implementation costs of $53–350 million.^10^ In other words, the cost of compliance is 11–172 times less than the health costs that individuals and health programs would likely bear if the standard were not tightened.^11^

One of industry’s greatest concerns about the new regulations is the uncertainty in getting permits for new or expanded facilities in counties that are barely compliant, Gershman says. Partly because of limitations with current modeling efforts, that uncertainty could linger through 2014, when the EPA, working with state, tribal, and local governments, is expected to finalize decisions on which counties violate the standard. “Our focus will be on working with the EPA to get more comprehensive modeling guidance,” she says.

Georges Benjamin, executive director of the American Public Health Association, would like to have seen all the other PM_2.5_ and PM_10_ standards made more stringent, too, but he speculates the agency won’t go that route, despite existing scientific support for doing so, until pollution control technology improves in capability and/or cost. “You can’t ask industry to do something impossible,” he says. “There’s a tradeoff there.”
