# Erratum: “New Primary Standard Set for Fine Particulate Matter Pollution” [121(3):A74 (2013)]

**DOI:** 10.1289/ehp.121-a113b

**Published:** 2013-04-01

In a typographical error, the March 2013 News article “New Primary Standard Set for Fine Particulate Matter Pollution” (Environ Health Perspect 121:A74; doi: 10.1289/ehp.121-a74) incorrectly expressed the 24-hour primary standard and annual and 24-hour secondary standards for PM_2.5_sub> in milligrams per cubic meter. The standards should have been expressed as micrograms per cubic meter.

*EHP* regrets the errors.

